# Development of a real-time cattle lameness detection system using a single side-view camera

**DOI:** 10.1038/s41598-024-64664-7

**Published:** 2024-06-14

**Authors:** Bo Bo Myint, Tsubasa Onizuka, Pyke Tin, Masaru Aikawa, Ikuo Kobayashi, Thi Thi Zin

**Affiliations:** 1 Graduate School of Engineering, University of Miyazaki, Miyazaki, 889-2192 Japan; 2 Organization for Learning and Student Development, University of Miyazaki, Miyazaki, 889-2192 Japan; 3 Sumiyoshi Livestock Science Station, Field Science Center, Faculty of Agriculture, University of Miyazaki, Miyazaki, 889-0121 Japan

**Keywords:** Computer science, Information technology

## Abstract

Recent advancements in machine learning and deep learning have revolutionized various computer vision applications, including object detection, tracking, and classification. This research investigates the application of deep learning for cattle lameness detection in dairy farming. Our study employs image processing techniques and deep learning methods for cattle detection, tracking, and lameness classification. We utilize two powerful object detection algorithms: Mask-RCNN from Detectron2 and the popular YOLOv8. Their performance is compared to identify the most effective approach for this application. Bounding boxes are drawn around detected cattle to assign unique local IDs, enabling individual tracking and isolation throughout the video sequence. Additionally, mask regions generated by the chosen detection algorithm provide valuable data for feature extraction, which is crucial for subsequent lameness classification. The extracted cattle mask region values serve as the basis for feature extraction, capturing relevant information indicative of lameness. These features, combined with the local IDs assigned during tracking, are used to compute a lameness score for each cattle. We explore the efficacy of various established machine learning algorithms, such as Support Vector Machines (SVM), AdaBoost and so on, in analyzing the extracted lameness features. Evaluation of the proposed system was conducted across three key domains: detection, tracking, and lameness classification. Notably, the detection module employing Detectron2 achieved an impressive accuracy of 98.98%. Similarly, the tracking module attained a high accuracy of 99.50%. In lameness classification, AdaBoost emerged as the most effective algorithm, yielding the highest overall average accuracy (77.9%). Other established machine learning algorithms, including Decision Trees (DT), Support Vector Machines (SVM), and Random Forests, also demonstrated promising performance (DT: 75.32%, SVM: 75.20%, Random Forest: 74.9%). The presented approach demonstrates the successful implementation for cattle lameness detection. The proposed system has the potential to revolutionize dairy farm management by enabling early lameness detection and facilitating effective monitoring of cattle health. Our findings contribute valuable insights into the application of advanced computer vision methods for livestock health management.

## Introduction

Video analysis and a Siamese attention model (Siam-AM) offer a potential solution for tracking all four legs of cattle and detecting lameness in dairy herds. The process involves feature extraction, applying attention weighting, and comparing similarities to achieve precise leg tracking^1^. Dairy cattle lameness significantly impacts their health and well-being, leading to decreased milk production, extended calving intervals, and increased costs for producers^2–4^. Research suggests associations between low body condition scores, hoof overgrowth, early lactation, larger herd sizes, and higher parity with increased odds of lameness in stall-housed cattle. However, data retrieval challenges and limited study comparability highlight the need for robust evidence to develop effective intervention strategies^5^. Early identification of lameness allows for more cost-effective treatment, making automatic detection models an optimal solution to reduce expenses^6^. Additionally, lameness reduction offers the potential for decreased recurrence of past events and improved cattle body condition scores^7^. Prompt detection and management of lameness are crucial for the sustainable growth of the dairy industry. However, manual detection becomes increasingly challenging as dairy farming expands^8^.

Studies investigating the intra- and inter-rater reliability of lameness assessment in cattle using locomotion scores, both live and from video, have shown that experienced raters demonstrate higher reliability when scoring from video. This suggests video observation is an acceptable method for lameness assessment, regardless of the observer's experience^9^. Lameness poses a significant welfare concern for dairy cattle, leading to various gait assessment methods. While subjective methods lack consistency, objective ones require advanced technology. This review evaluates the reliability, validity, and interplay of gait assessment with cattle factors, hoof pathologies, and environmental conditions^10^. This study employs a dynamic stochastic model to assess the welfare impact of various foot disorders in dairy cattle. Results highlight the significant negative welfare effects, with digital dermatitis having the highest impact, followed by subclinical disorders like sole hemorrhages and interdigital dermatitis. This study emphasizes the previously underappreciated impact of subclinical foot disorders and highlights the importance of considering pain intensity and clinical conditions in welfare assessments and management strategies^11^.

Clinical lameness negatively affects milk yield and reproductive performance in cattle^12,13^. Traditionally, farmers have relied on visual observation through locomotion scoring for diagnosing lameness^14^. However, this method is resource-intensive, time-consuming, and relies on qualitative assessments of gait and posture changes^15^. Additionally, individual cattle variations and dynamic movement pose challenges^16^. Claw lesions, both infectious and non-infectious, remain the primary cause of lameness in cattle^17^. Recent research has explored diverse characteristics of body motion to describe and detect lameness^18–20^. One expert survey assigned weights to various gait factors, finding that analyzing cattle leg swing holds potential for lameness determination, given that most indicators relate to walking performance^21^. Identifying lameness remains a challenge in the dairy sector due to its impact on reproductive efficiency, milk production, and culling rates^22^. It ranks as the third most economically impactful disease in cattle, following fertility and mastitis^23^. Early detection is crucial, leading to reduced antibiotic use and enhanced milk yield^19,24^. To address these challenges, one study proposed a method that identifies deviations from normal cattle gait patterns using sensors like accelerometers to capture walking speed data and integrate it into a prediction model^25^.

However, this contact-based approach may initially stress cattle unaccustomed to the equipment. Implementing it on a large scale would also increase labor and equipment costs. Overcoming these limitations, some researchers propose non-contact methods using monitoring cameras on farms. By modeling time and space for cattle tracking, they achieved relatively accurate results. However, this method lacked environmental robustness, as changes in conditions could negatively impact the algorithm's performance and lead to subpar results in long-term detection^26^. In recent years, researchers have shown significant interest in object detection using convolutional neural networks (CNNs)^27,28^ and feature classification based on recurrent neural networks (RNNs)^29–32^. One study highlighted that deep learning-based object detection and recognition methods can overcome the limitations of manual design features, which often lack diversity^33^. These approaches hold substantial promise for cattle lameness detection. By leveraging CNNs for cattle object detection and RNNs for feature extraction, valuable and continuous data can be effectively extracted, offering significant potential for cattle lameness detection. Studies have shown a correlation between the degree of curvature in a lame animal's back and the severity of lameness^19,20^.

Utilizing automatic back posture measurements in daily routines allows for individual lameness classifications. Another study proposed a method based on consecutive 3D-video recordings for automatic detection of lameness^34^. This study focuses on exploring the causes of lameness and early detection methods. Prior to classification, the system detects the cattle region using an instance segmentation algorithm to extract the region's mask value, which is crucial for feature extraction. This study combines image processing techniques and deep learning methods for detection and tracking. The extracted feature values are classified using popular machine learning algorithms like support vector machines and random forests. Our work utilizes the well-regarded Mask R-CNN instance segmentation algorithm for cattle region extraction. Subsequently, we employ Intersection-over-Union (IoU) in conjunction with frame-holding and entrance gating mechanisms to identify and track individual cattle. Finally, image processing techniques are leveraged to extract distinct features from these regions, enabling the calculation of cattle lameness. The primary contributions of our paper are as follows:

(i) Accurate detection and instance segmentation of cattle located behind the frame or within covered areas remain critical challenges. This work seeks to address these complexities.
(ii) To assess the efficacy of our custom-trained Mask R-CNN model, we performed a comparative analysis with the state-of-the-art YOLOv8 algorithm.
(iii) For cattle tracking, we developed a light, customized algorithm that leverages IoU calculations combined with frame-holding and entrance gating mechanisms.
(iv) This work proposes a novel approach to assess cattle lameness by analyzing the variance in movement patterns. We achieve this by calculating the three key points on the back curvature of the cattle, providing a new metric for lameness evaluation.
(v) Comparing individual cattle lameness scores directly lack robustness. Instead, calculating the probabilities across all frames from each result folder (obtained from the cattle tracking phase) provides a more robust approach.
(vi) The integration of cattle detection, tracking, feature extraction, and lameness calculation enables real-time cattle lameness detection.
(vii) Early lameness detection in cattle remains a significant challenge, with most research focusing on differentiating between non-lame (level 1) and mildly lame (levels 2 and 3) animals.

## Related work

In our Visual Information Focusing on animal welfare and technological advancements, the Visual Information Lab at Miyazaki University is currently engaged in the ongoing research and development of several key areas related to cattle management. Specifically, our focus lies on the following three systems such as Cattle Lameness Level Classification System, Cattle Body Condition Classification System, Cattle Mounting Detection System. For each of these systems, our research efforts encompass the development of robust detection, tracking and identification methodologies^35–40^, specifically tailored to capture the relevant features and characteristics of the cattle under observation. Additionally, we are diligently working on devising efficient and accurate classification algorithms that will aid in categorizing the identified cattle attributes according to their respective criteria within each system^41^. Our goal is to contribute to advancements in cattle management practices by providing innovative and reliable technological solutions in this domain. Lameness in cattle is of utmost importance as it can have a significant impact on animal welfare, production efficiency, and economic losses in the livestock industry.

Early and accurate detection of lameness is essential to ensure timely intervention and appropriate treatment for affected animals. In recent years, cattle lameness classification has garnered considerable attention in the field of precision livestock farming. Researchers have explored various approaches to achieve accurate and timely identification of cattle lameness using advanced technologies and innovative methods. Several research endeavors have contributed significantly to the domain of cattle lameness detection and body movement variability. In the context of detecting lameness in dairy cattle, several research studies have explored intelligent methods that leveraged advanced computer vision techniques, specifically Mask-RCNN, to extract the region of interest encompassing the dairy cattle. By utilizing features derived from head bob patterns, the authors successfully identified potential signs of lameness in the cattle movement^42^. Several research studies have been conducted to address this critical issue, focusing on the development of accurate and efficient lameness detection systems.

To detect lameness in cattle,^43^ proposed a computer vision-based approach that emphasizes the lameness detection of dairy cattle by implementing an intelligent visual perception system using deep learning instance segmentation and identification to provide a cutting-edge solution that effectively extracts cattle regions from complex backgrounds. The^44^ study introduces an in-parlor scoring (IPS) technique and compares its performance with LS in pasture-based dairy cattle. IPS indicators, encompassing shifting weight, abnormal weight distribution, swollen heel or hock joint, and overgrown hoof, were observed, and every third cattle was scored. The findings suggest that IPS holds promise as a viable alternative to LS on pasture-based dairy farms, presenting opportunities for more effective lameness detection and management in this setting. The lameness monitoring algorithm based on back posture values derived from a camera by fine-tuning deviation thresholds and the quantity of historical data used is developed in^45^. The paper introduces a high-performing lameness detection system with meaningful historical data utilization in deviation detection algorithms.

The^46^ study proposes a novel lameness detection method that combines machine vision technology with a deep learning algorithm, focusing on the curvature features of dairy cattle’s' backs. The approach involves constructing three models: Cattle's Back Position Extraction (CBPE), Cattle's Object Region Extraction (CORE), and Cattle's Back Curvature Extraction (CBCE). A Noise + Bilateral Long Short-term Memory (BiLSTM) model is utilized to predict the curvature data and match the lameness features. The^47^ introduces developing a computer vision system using deep learning to recognize individual cattle in real-time, track their positions, actions, and movements, and record their time history outputs. The YOLO neural network, trained on cattle coat patterns, achieved a mean average precision ranging from 0.64 to 0.66, demonstrating the potential for accurate cattle identification based on morphological appearance, particularly the piebald spotting pattern. Data augmentation techniques were employed to enhance network performance and provide insights for efficient detection in challenging data acquisition scenarios involving animals. The authors from^48^ present an end-to-end Internet of Things (IoT) application that leverages advanced machine learning and data analytics techniques for real-time cattle monitoring and early lameness detection. Using long-range pedometers designed for dairy cattle, the system monitors each cattle's activity and aggregates the accelerometric data at the fog node. The development of an automatic and continuous system for scoring cattle locomotion, detecting and predicting lameness with high accuracy and practicality is represented in^49^. Using computer vision techniques, the research focuses on analyzing leg swing and quantifying cattle movement patterns to classify lameness. By extracting six features related to gait asymmetry, speed, tracking up, stance time, stride length, and tenderness, the motion curves were analyzed and found to be nearly linear and separable within the three lameness classes.

In^50^, the authors presented a pioneering method that harnessed depth imaging data to assess the variability of cattle body movements as an indicator of lameness. Their framework involved the development of an operational simulation model, integrating Monte Carlo simulation with prevalent probability distribution functions, including uniform, normal, Poisson, and Gamma distributions. By leveraging these techniques, the researchers were able to analyze the influence of key factors on cattle lameness status. The depth video camera-based system to detect cattle lameness from a top view position is researched in^51^. In their method, they extracted depth value sequences from the cattle body region and calculated the greatest value of the rear cattle area. By calculating the average of the maximum height values in the cattle backbone area, they created a feature vector for lameness classification. The authors then employed Support Vector Machine (SVM) to classify cattle lameness based on the computed average values. Their findings demonstrated the potential of using depth video cameras and SVM for efficient lameness detection.

This research^52^ communication explores the correlation between lameness occurrence and body condition score (BCS) by employing linear mixed-effects models to assess the relationship between BCS and lameness. The study revealed that the proportion of lame cattle increased with decreasing BCS, but also with increasing BCS. The likelihood of lameness was influenced by the number of lactations and decreased over time following the last claw clipping. This suggests the importance of adequate body condition in preventing lameness, while also raising questions about the impact of over conditioning on lameness and the influence of claw trimming on lameness assessment. The system^53^ uses computer vision and deep learning techniques to accurately analyze the posture and gait of each cattle within the camera's field of view. The tracking of cattle as they move through the video sequence was performed using the SORT algorithm. The features obtained from the pose estimation and tracking were combined using the CatBoost gradient boosting algorithm. The system's accuracy was evaluated using threefold cross-validation, including recursive feature elimination. Precision was assessed using Cohen's kappa coefficient, and precision and recall were also considered.

Building upon the pioneering work of^54^ represents a pioneering use of deep learning-based gait reconstruction and anomaly detection for early lameness detection, leveraging the portability and real-time capabilities of wearable gait analysis to enhance animal welfare, our proposed system prioritizes a more natural approach. We aim to minimize stress on cattle and avoid altering their environment. This focus on natural interaction positions our system as a significant advancement for animal welfare and management practices in the dairy industry. This work utilizes Mask R-CNN, a popular instance segmentation algorithm implemented by Detectron 2, and the state-of-the-art YOLOv8 for cattle detection. To optimize feature analysis for individual cattle across frames, we consider additional features and leverage cattle tracking with a simple and efficient IoU calculation and frame-holding logic. Our proposed cattle lameness detection methodology employs a three-point back curvature approach that integrates movement variances to calculate lameness levels. The “Methodology” section describes the specific technologies utilized in our proposed system.

## Methodology

**Figure 1:**
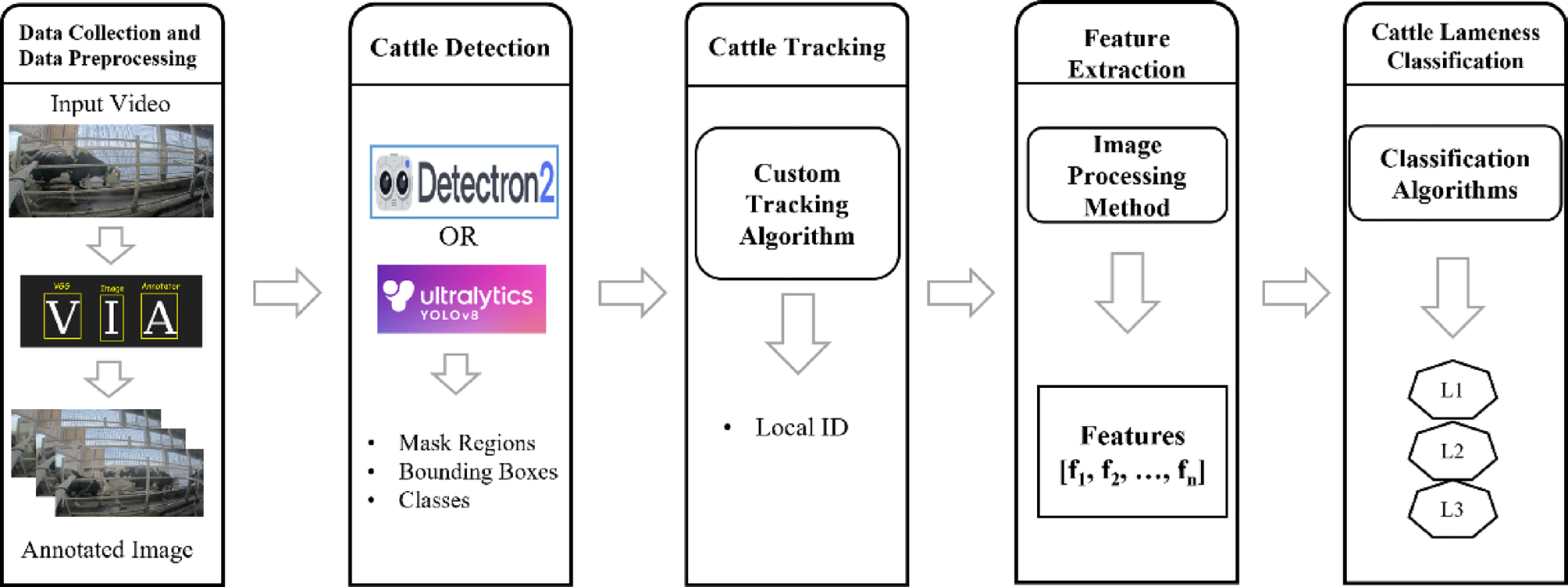
Overall pipeline of the proposed cattle lameness classification system: (1) data collection and data preprocessing (2) cattle detection (3) cattle tracking (4) feature extraction (5) cattle lameness classification.

This study proposes a novel system for automatically calculating cattle lameness from farm video footage. As illustrated in Fig. 1, the system employs a five-stage pipeline: (1) Data Collection and Data Preprocessing, (2) Cattle Detection, (3) Cattle Tracking, (4) Feature Extraction, and (5) Cattle Lameness Classification. During the Data Collection and Data Preprocessing stage, videos are segmented into individual frames for subsequent analysis. Dedicated annotation tools are then employed to manually label frames, generating ground truth data for training the cattle detection model. The Detection stage leverages annotated data to train two object detection models for comparative analysis: a Mask R-CNN detector implemented within the Detectron2 framework^27^ and the state-of-the-art YOLOv8 model. Both models are tasked with localization and classification of cattle within each frame, generating bounding boxes, mask regions, and class labels for each detected animal.

Subsequently, the Tracking stage leverages the bounding box information to track individual cattle traveling within a defined area. Each tracked animal is assigned a unique Local ID for subsequent analysis. In the Feature Extraction stage, a diverse set of features (*F*_*1*_*, F*_*2*_*, …, F*_*n*_) are calculated for each tracked individual. These features, carefully chosen to capture relevant movement characteristics, serve as input for the subsequent lameness classification stage. Finally, the Classification stage employs various machine learning algorithms to categorize cattle into three groups: early lameness levels (2 and 3) and no lameness (level 1). The classification results are stored daily, providing valuable information for monitoring herd health, and enabling early intervention for lameness management. Overall, this proposed system offers a novel approach for utilizing farm video data to gain valuable insights into cattle well-being by facilitating the early detection and management of lameness.

### Data collection and data preprocessing

**Figure 2:**
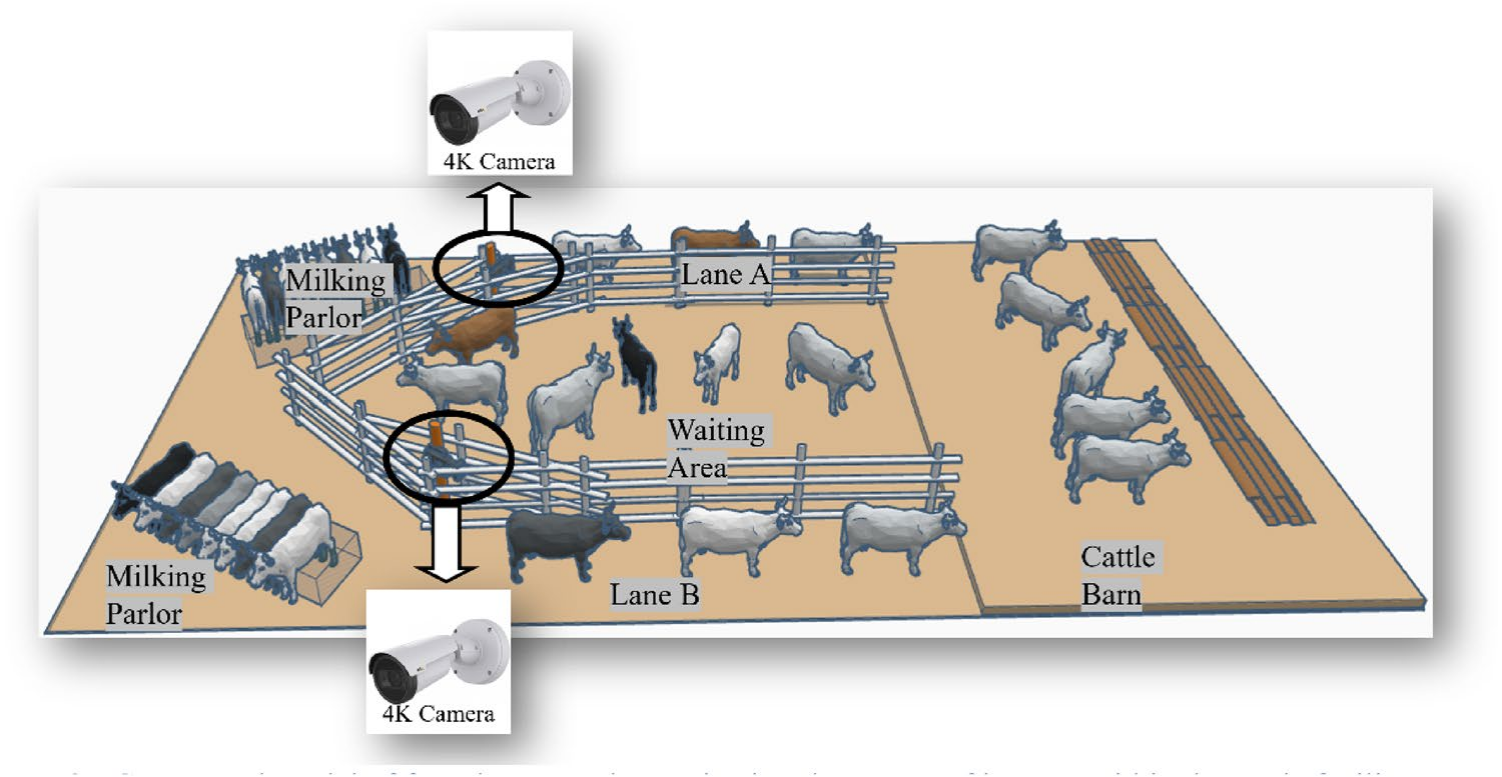
Conceptual model of farm layout and organization: key areas of interest within the cattle facility include milking parlors, designated lanes (lane A and lane B), a waiting area, and cattle barn.

**Figure 3:**
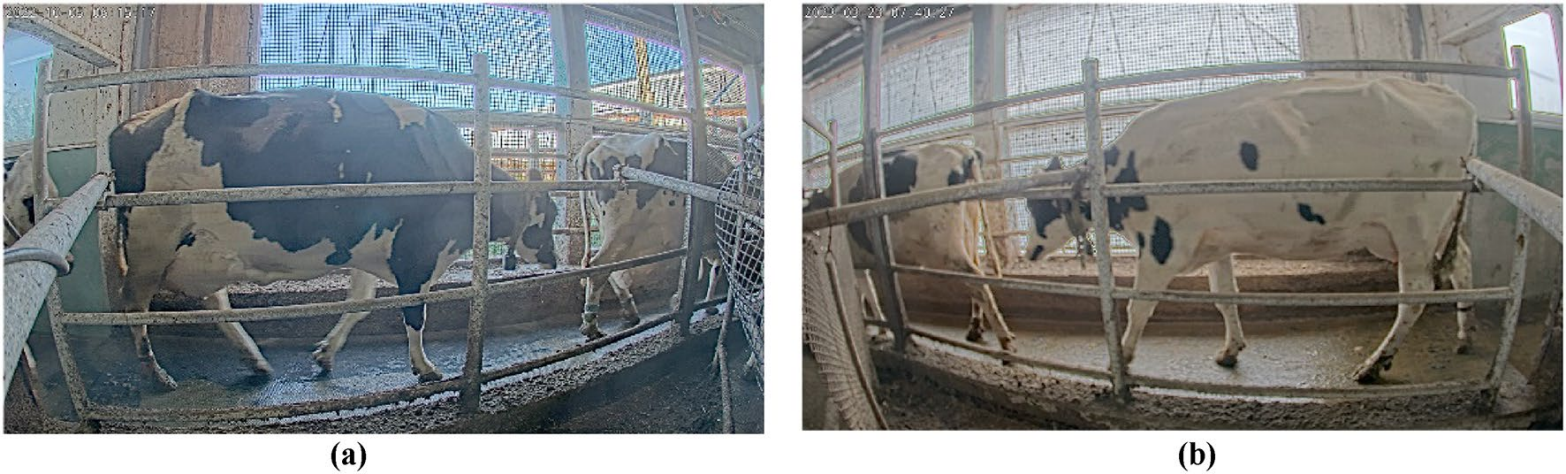
Sample Image from cattle farm (**a**) cattle lane A (**b**) cattle lane B.

This study utilized a cattle dataset collected at the Hokuren Kunneppu Demonstration Farm in Hokkaido, Japan. The farm features two distinct cattle passing lanes leading from the Cattle Barn to the Milking Parlor as shown in Fig. 2. Between these lanes lies cattle waiting area where groups of eight or seven cattle await their turn to enter the milking parlor. To facilitate data collection, a single camera was strategically positioned at the starting point of each lane, enabling comprehensive monitoring and analysis of cattle behavior and movement along the pathways. Figure 3a,b depict Lane A and Lane B, respectively.

#### Data collection

**Figure 4:**
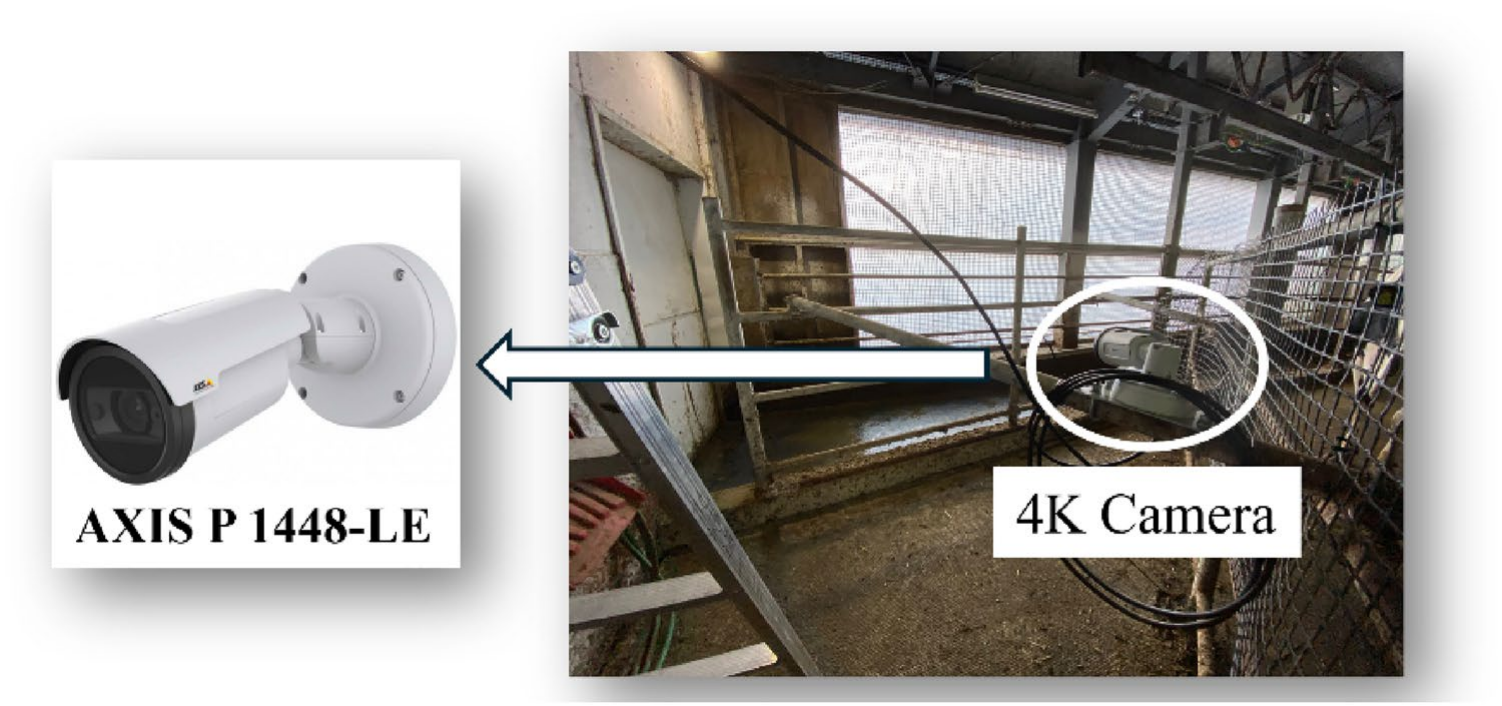
Camera configuration for monitoring lane A in the cattle Farm and camera type of 4K camera usage in cattle farm.

**Figure 5:**
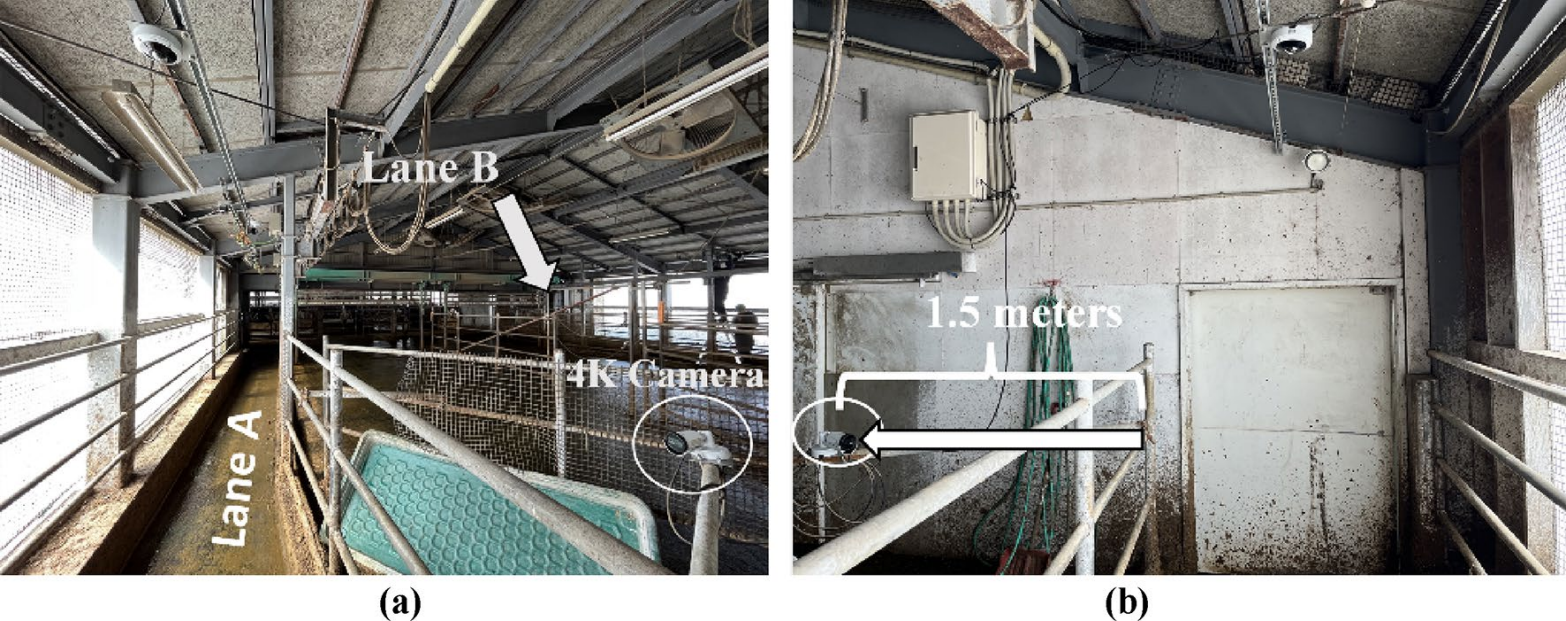
Camera installation (**a**) 4K camera on lane A (**b**) front-view from 4K camera installed.

This study utilized two AXIS P 1448-LE 4K cameras (Fig. 4) strategically positioned at the starting points of the two cattle lanes connecting the cattle barn to the milking parlor (Fig. 5a,b). These cameras captured video recordings at a frame rate of 25 frames per second and an image resolution of 3840 × 2160. During preprocessing, a subset of images was extracted from the video at a reduced frame rate (13 fps) and resolution (1280 × 720) within the region of interest, optimizing data processing and resource allocation efficiency. The primary focus of the study was on Lane A, although Lane B was also present (Fig. 3). Camera recordings were conducted during specific time intervals (5 am–8 am and 2 pm–5 pm) to coincide with natural cattle movement patterns through the designated lanes. This approach ensured data collection occurred without the need for additional handling or disruptions to the cattle's daily routine, minimizing stress and maintaining their well-being.

#### Data preprocessing

**Figure 6:**
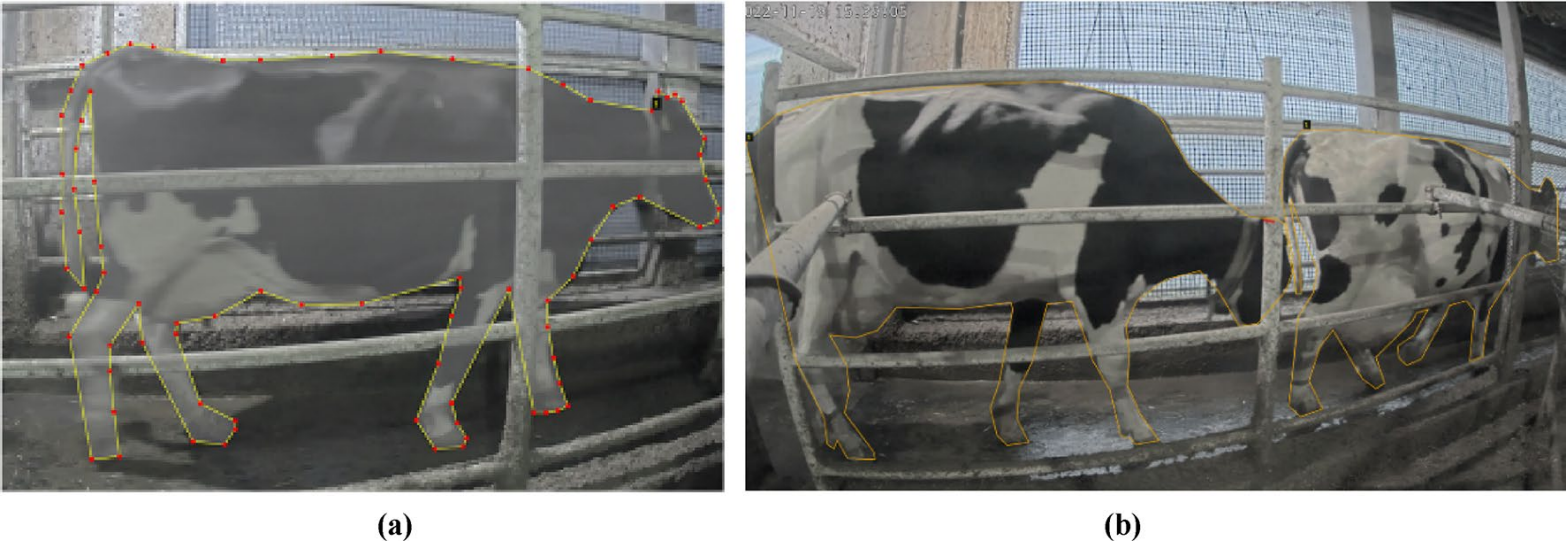
(**a**), (**b**) Sample annotations of cattle region by using VGG image annotator.

For data preprocessing in cattle region detection, we employed VGG Image Annotator (VIA)^55^, a simple and standalone annotation tool for images. VIA facilitated manual annotation, allowing us to mark cattle regions using various shapes, such as box, polygon, and key points (skeleton), among others. In our study, we utilized the polygon shape to annotate cattle regions, where the boundaries of the cattle region were marked with pixel points. These grouped pixels were then labeled as "cattle," as depicted in Fig. 6a,b. Throughout the training process for cattle detection, we utilized a single class named "cattle." The use of VIA provided a lightweight and installation-free solution for efficient data annotation and preparation.

To prepare the cattle detection training dataset, we manually annotated 5458 Instances across three distinct dates: July 4th, September 30th, and November 19th, and for testing dataset we collected 247,250 instances from January 3rd, 5th, 6th, 7th, 10th, 11st, 12nd, 13rd, 14th, 23rd, 24th, 25th, 26th, 27th, 28th and 29th as detailed in Table 1. The January dataset presented the most significant challenge for cattle detection due to the combined effects of the winter season and camera setup environment. Specifically, the presence of smoke in the environment and proximity of cattle seeking warmth during cooler temperatures hindered accurate detection. Recognizing the crucial role of data quality in model performance, we implemented a multi-step approach to optimize the dataset: (1) Duplicate Removal: We meticulously identified and eliminated duplicate images, minimizing redundancy, and alleviating unnecessary computational strain during training. (2) Blur Mitigation: Recognizing the detrimental impact of blurred images on detection accuracy, we meticulously filtered out any exhibiting noticeable blurring, thereby fostering a dataset of enhanced quality. (3) Noise Reduction: Images afflicted by excessive noise, such as pixelation or distortion, were meticulously excluded, ensuring the inclusion of noise-free images that bolster the model's robustness. (4) Relevant Data Extraction: As our focus was on cattle detection, images devoid of cattle passing lanes or lacking pertinent cattle-related information were meticulously excluded, guaranteeing the training dataset comprised solely of high-quality, diverse, and relevant imagery, ultimately optimizing the detection model's performance and effectiveness.

**Table 1:** Dataset used for training and testing of cattle detection.

Type of training	Date	Duration	Number of instances
Training	4th July 2022	Morning, evening	3000
	30th September 2022	Morning, evening	480
	19th November 2022	Morning, evening	1978
Testing	3rd January 2023	Morning, evening	17,322
	5th January 2023	Morning, evening	22,194
	6th January 2023	Morning	5059
	7th January 2023	Morning	5106
	10th January 2023	Morning, evening	20,720
	11st January 2023	Morning, evening	11,257
	12nd January 2023	Morning, evening	16,274
	13rd January 2023	Morning, evening	24,538
	14th January 2023	Morning	6074
	23rd January 2023	Morning, evening	18,579
	24th January 2023	Morning, evening	23,684
	25th January 2023	Morning, evening	15,596
	26th January 2023	Morning, evening	14,998
	27th January 2023	Morning, evening	12,934
	28th January 2023	Morning	15,619
	29th January 2023	Morning, evening	17,296

### Cattle detection

In recent years, object detection has gained significant attention in the field of research, mainly due to the advancements made by machine learning and deep learning algorithms. Object detection involves precisely locating and categorizing objects of interest within images or videos. To achieve this, the positions and boundaries of the objects are identified and labeled accordingly. Presently, state-of-the-art object detection methods can be broadly classified into two main types: one-stage methods and two-stage methods. One-stage methods prioritize model speed and efficiency, making them well-suited for real-time applications. Some notable one-stage methods include the Single Shot multibox Detector (SSD)^56^, You Only Look Once (YOLO)^57^, and RetinaNet^58^. On the other hand, two-stage methods are more focused on achieving high accuracy. These methods often involve a preliminary region proposal step followed by a detailed classification step. Prominent examples of two-stage methods include Faster R-CNN^59^, Mask R-CNN^60^, and Cascade R-CNN^61^. The choice between one-stage and two-stage methods depends on the specific requirements of the application. While one-stage methods are faster and more suitable for real-time scenarios, two-stage methods offer improved accuracy but may be computationally more intensive. As object detection continues to evolve, researchers and practitioners are exploring a range of techniques to strike the right balance between speed and accuracy for various use cases. In this study, we evaluated two state-of-the-art and widely used object detection algorithms within our research domain. The first algorithm is Mask R-CNN, which belongs to the two-stage methods and is implemented using the powerful Detectron2 framework. Mask R-CNN has gained popularity due to its robustness and flexibility in handling complex detection tasks, particularly in instances where both bounding boxes and pixel-wise segmentation masks are required. The second algorithm under consideration is YOLOv8, which is currently one of the most widely adopted and popular object detection models. YOLOv8 is known for its efficiency and real-time capabilities, making it suitable for various applications. It excels in handling detection tasks with high accuracy while maintaining impressive speed, making it a preferred choice in many scenarios. By comparing these two state-of-the-art detection algorithms, we aimed to gain insights into their performance and applicability within our research domain. The evaluation will help us determine which algorithm better suits our specific requirements and contributes to advancing object detection techniques in our field.

**Figure 7:**
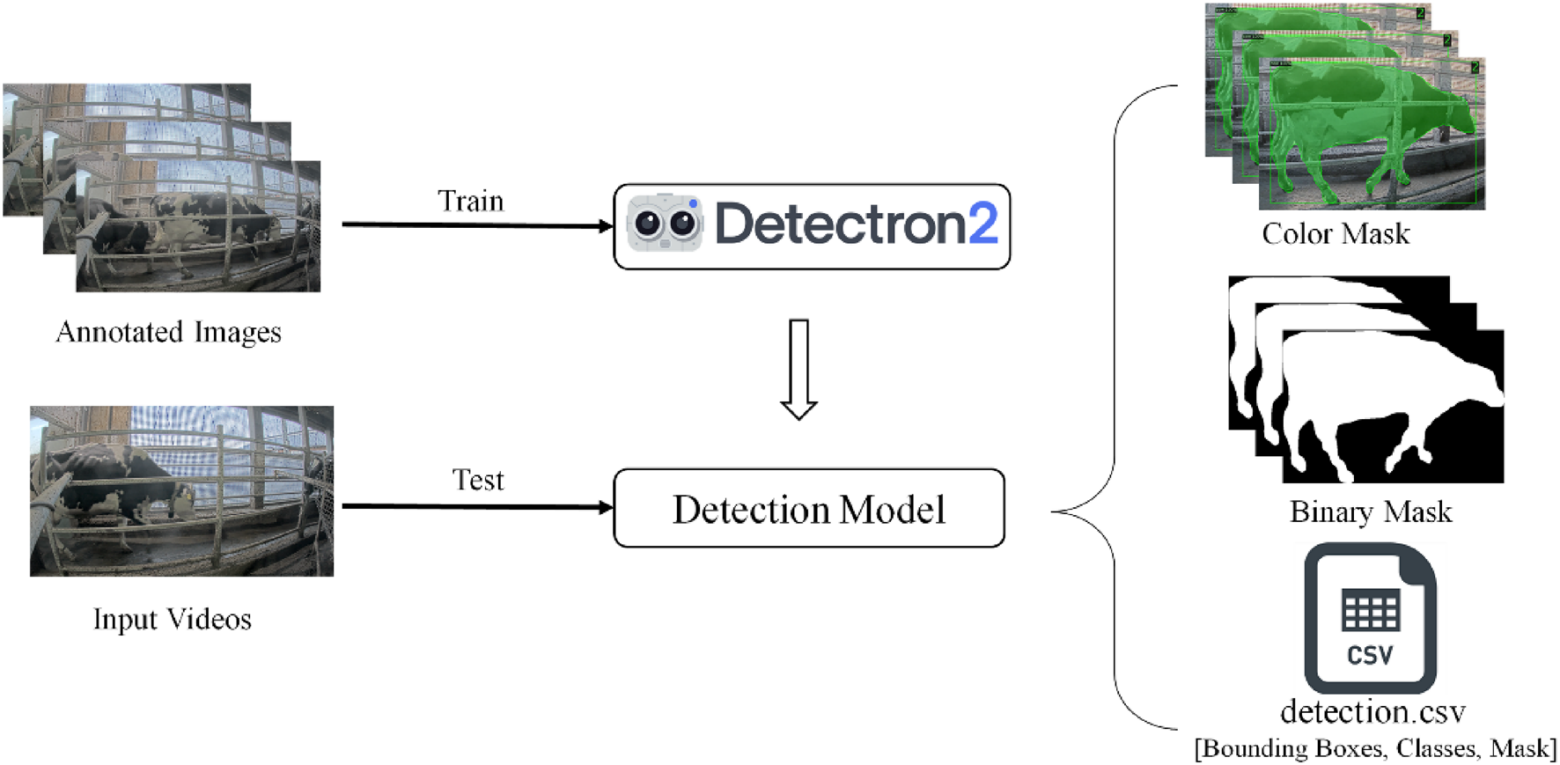
Architecture for cattle detection by using Detectron2: Custom training pipeline for our dataset.

Detectron2, developed by Facebook, is a state-of-the-art vision library designed to simplify the creation and utilization of object detection, instance segmentation, key point detection, and generalized segmentation models. Specifically, for object detection, Detectron2's library offers a variety of models, including RCNN, Mask R-CNN, and Faster R-CNN. RCNN generates region proposals, extracts fixed-length features from each candidate region, and performs object classification. However, this process can be slow due to the independent passing of CNN over each region of interest (ROI). Faster R-CNN architecture overcomes this limitation by incorporating the Region Proposal Network (RPN) and the Fast R-CNN detector stages. It obtains class labels and bounding boxes of objects effectively. Mask R-CNN, sharing the same two stages as Faster R-CNN, extends its capabilities by also generating class labels, bounding boxes, and masks for objects. Notably, Mask R-CNN demonstrates higher accuracy in cattle detection, as indicated by previous research. Therefore, the proposed system adopts Mask R-CNN to extract mask features specifically for cattle, as illustrated in Fig. 7. During the detection phase, the default predictor, COCO-Instance Segmentation, and Mask R-CNN with a 0.7 score threshold value (MODEL.ROI_HEADS.SCORE_THRESH_TEST) are utilized. The training process employs annotated images, while for testing, videos are fed into Detectron2 to obtain color mask, and binary mask images and detection results including bounding boxes, mask value of detected cattle region and cattle confidence score. The mask image is subsequently utilized in cattle detection calculations. Overall, by leveraging the capabilities of Mask R-CNN from Detectron2, the proposed system aims to achieve accurate and efficient cattle detection, offering valuable insights for cattle health monitoring and management.

**Figure 8:**
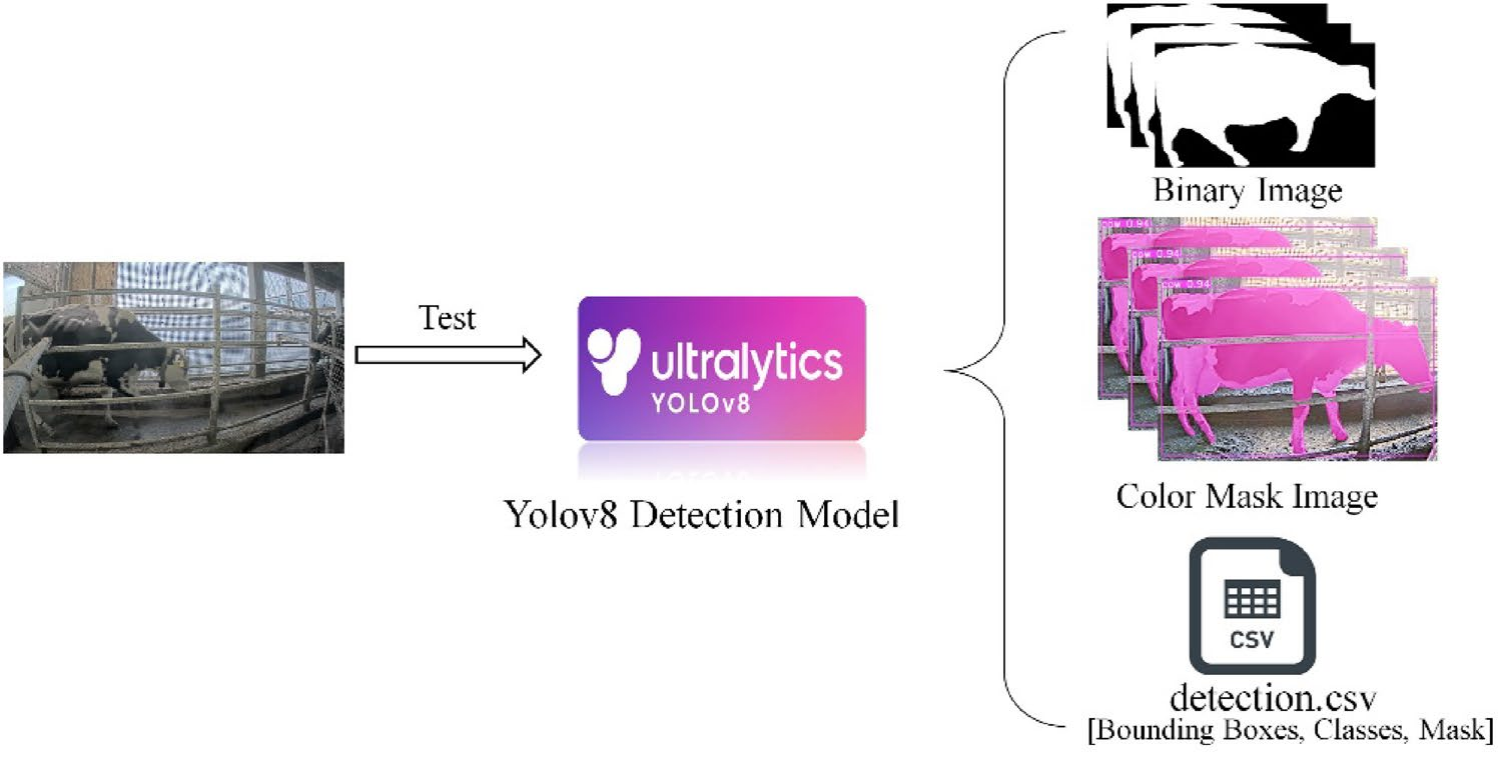
Architecture for cattle detection by using YOLOv8 base model.

Ultralytics YOLOv8 is a powerful and versatile object detection and instance segmentation model that is designed to be fast, accurate, and easy to use. It builds upon the success of previous YOLO versions and introduces new features and improvements to further boost performance and flexibility. Is applicable to a wide range of tasks, including object detection, tracking, instance segmentation, image classification, and pose estimation. There are five pre-trained models with different sizes available for instance segmentation: yolov8n (Nano), yolov8s (Small), yolov8m (Medium), yolov8l (Large), and yolov8x (Extra Large). Instance segmentation goes a step further than object detection by identifying individual objects in an image and segmenting them from the rest of the image. Instance segmentation is useful when you need to know not only where objects are in an image, but also what their exact shape is. In this research, we employ the YOLOv8x-seg model for cattle detection and segmentation. Unlike traditional methods, we do not rely on manual annotation or custom training specific to cattle. Instead, YOLOv8x-seg is applied directly to the task of cattle detection, leveraging its advanced capabilities and pretrained features. This approach, as shown in Fig. 8, allows us to achieve accurate and efficient cattle detection without the need for labor-intensive annotation processes or specialized training. By utilizing the powerful segmentation capabilities of YOLOv8x-seg, we can effectively identify and delineate cattle regions in images or videos, contributing to the overall success of the study.

### Cattle tracking

**Figure 9:**
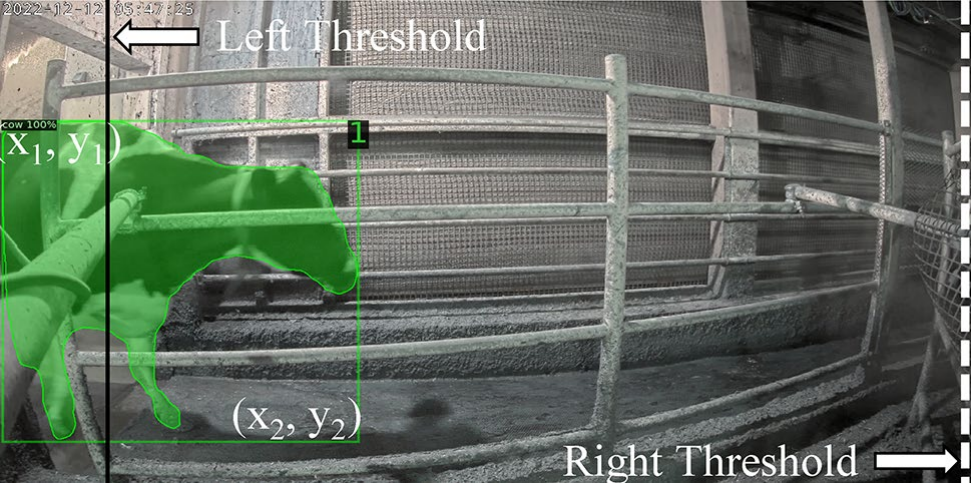
Cattle tracking logic (bounding box: [x_1_, y_1_, x_2_, y_2_], left threshold = entrance gate, right threshold = exit gate).

In this study, the movement of cattle through individual lanes necessitates precise individual tracking to enable accurate lameness calculation. Ensuring accurate cattle identification and storage is of paramount importance. The dataset's simplicity involves the traversal of just one or two cattle through the lanes. The system employs a designated region of interest, demarcated by the lines in Fig. 9, aligned with the camera settings. The tracking procedure features two vertically intersecting red lines within the frame. The left line represents the left threshold, and the corresponding right line serves as the right threshold. The amalgamation of computer vision, machine learning, and deep learning in recent years has yielded remarkable strides in tracking algorithms, leading to substantial improvements in accuracy.

**Algorithm 1:**
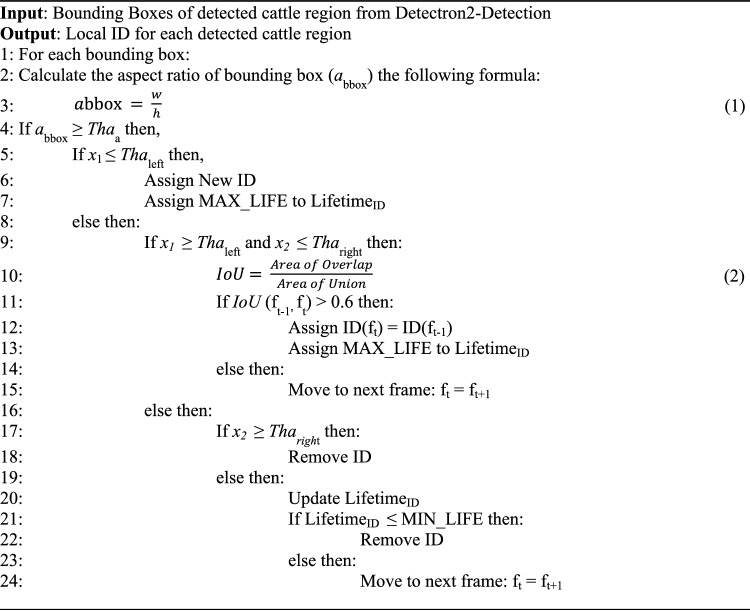
Customized tracking algorithm (CTA).

**Figure 10:**
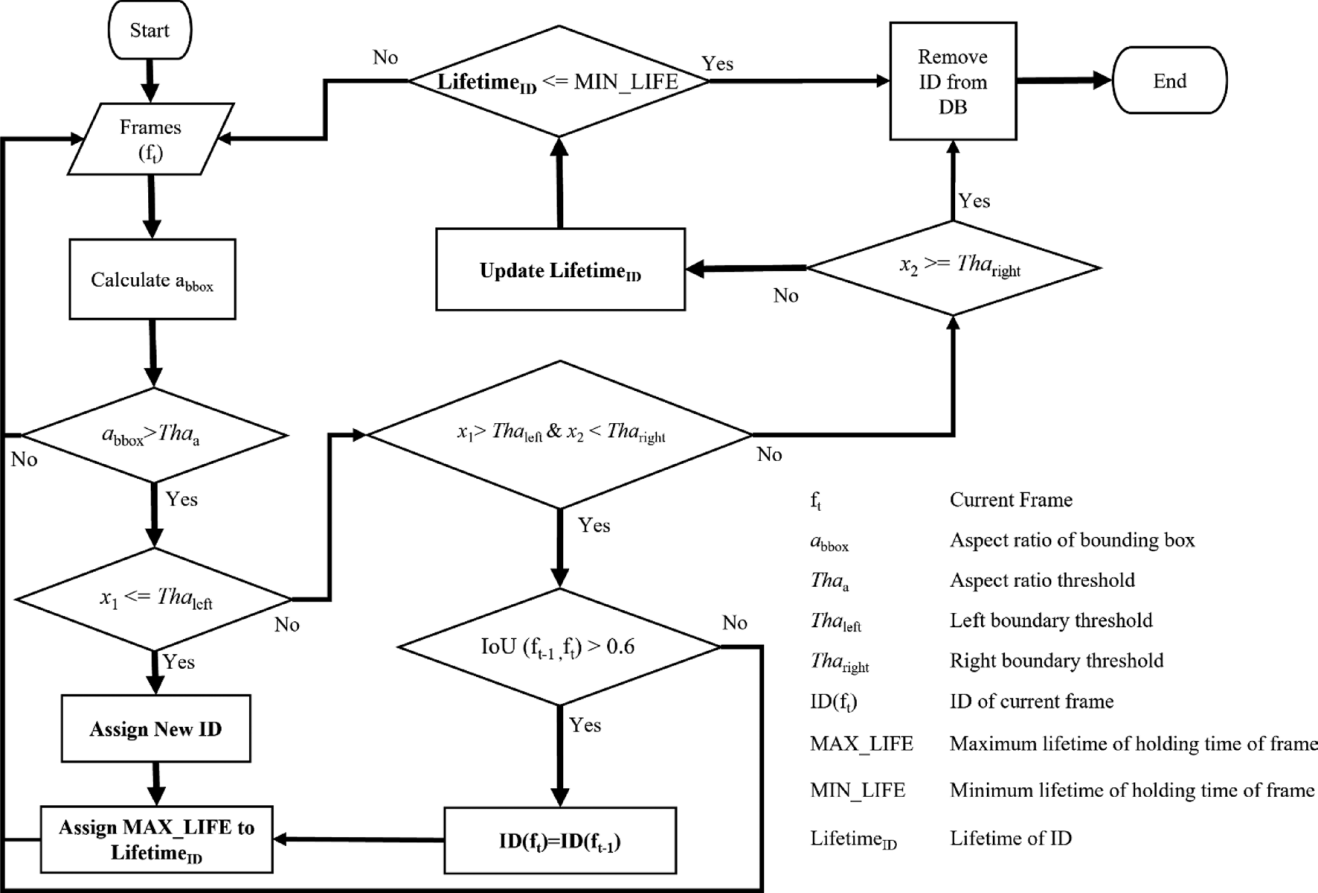
Automated cattle tracking process flow diagram.

In pursuit of cost-effectiveness and time efficiency, the research leveraged Intersection over Union (IoU) calculations for tracking, supplemented by the incorporation of individual ID lifetimes. This strategy, bolstered by Fig. 10, tackles challenges like missed detections and other detection intricacies. And Table 2 explains the variable use in tracking flowchart. This approach stands in contrast to conventional cattle tracking algorithms, offering simplicity, ease of manipulation, and minimal inference time. Tracking initiation occurs when a cattle's position precedes the left threshold and concludes upon the right side of the cattle's bounding box surpassing the right threshold. Within this tracking phase, the assignment of a cattle's Local ID transpires prior to traversing the left threshold, with the resulting Local ID stored in the Temporal Database. This meticulous tracking method ensures accuracy and efficiency, thereby contributing to the study's comprehensive monitoring and analysis objectives.

**Table 2:** Definitions of key variables for cattle tracking analysis.

Symbols	Description
f_t_	Current frame
*a*_bbox_	Aspect ratio of bounding box
*Tha*_a_	Aspect ratio threshold
*Tha*_left_	Left boundary threshold
*Tha*_right_	Right boundary threshold
ID(f_t_)	ID of current frame
MAX_LIFE	Maximum lifetime of holding time of frame
MIN_LIFE	Minimum lifetime of holding time of frame
Lifetime_ID_	Lifetime of ID

The provided Algorithm 1 outlines a comprehensive approach to assigning Local IDs to detected cattle regions based on Bounding Boxes obtained from the Detectron2-Detection process. The algorithm focuses on accurate and reliable tracking of individual cattle as they pass through lanes. Beginning with calculating the aspect ratio of each bounding box, the algorithm applies specific conditions to determine the appropriate assignment of Local IDs. It considers factors such as the position of bounding boxes relative to defined thresholds and employs techniques like Intersection over Union (IoU) calculations to ensure consistency in the tracking process. Moreover, the algorithm introduces the concept of lifespan (LifetimeID) for each assigned ID, allowing for refined management of cattle tracking.

By combining these strategies, the algorithm contributes to enhancing the accuracy and effectiveness of cattle tracking, thus enabling the monitoring of cattle behavior and health in various contexts. The local ID associated with a cattle's tracking information is systematically managed to ensure accurate and up-to-date records. This process involves the removal of the local ID from the database under certain circumstances. Specifically, when the horizontal position (*x*_*2*_) of the bounding box aligns with the right threshold of the frames in Fig. 10, indicating that the cattle have completed their passage through the designated lane, the local ID is removed. Additionally, if cattle exit the lane or remain undetected for ten consecutive frames within the designated Region of Interest (ROI), the local ID is also removed from the database. Careful management of local IDs ensures that the tracking records remain aligned with the real-time movements and presence of cattle within the monitored area, contributing to accurate and reliable tracking outcomes.

**Figure 11:**
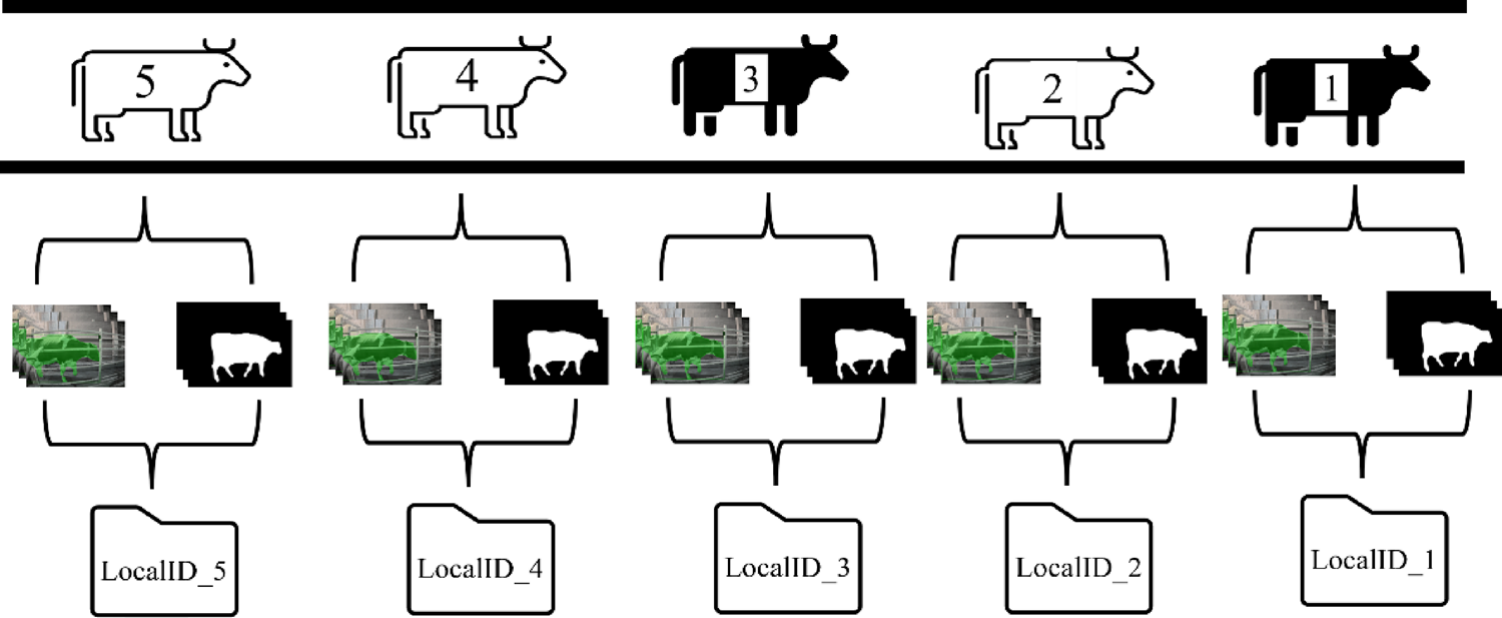
Cattle tracking summary: local IDs and associated results.

Upon the culmination of the tracking process, the cattle are systematically organized based on their unique cattle IDs, creating distinct folders as depicted in Fig. 11. Each of these folders contains a collection of essential images and binary masks. These include binary images that accentuate the delineation of the cattle, color mask images that highlight specific features, original binary masks that capture the raw attributes, and the unaltered original images of the cattle. This meticulous organization ensures that each cattle's data is readily accessible and preserved, contributing to the efficient analysis and retrieval of pertinent information for further research and examination.

### Feature extraction

**Figure 12:**
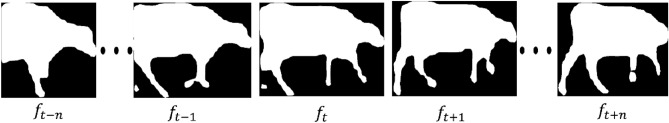
Segmentation of cattle in consecutive frames for feature extraction (f_t_ = current frame).

A sequencies of frames [*f*_*t-n*_, …, *f*_*t-*1_, *f*_*t*_, *f*_*t*+1_, *f*_*t*+*n*_] of each identification of cattle was captured by camera. Using Frame(*f*_*t-n*_) might be suitable for extracting Feature 1 (*F*_1_), but for the other features, it could present problems. The frame (*f*_*t*_) shown in Fig. 12 was the optimal choice for extracting all features, but there may be instances where the cattle head overlaps with its body. Utilizing features from all frames could result in some regions of the cattle not contributing useful information for lameness classification. Following the completion of cattle detection and tracking, individual cattle were sorted by their respective local IDs. From these sorted cattle, binary masks representing the cattle regions are obtained.

**Figure 13:**
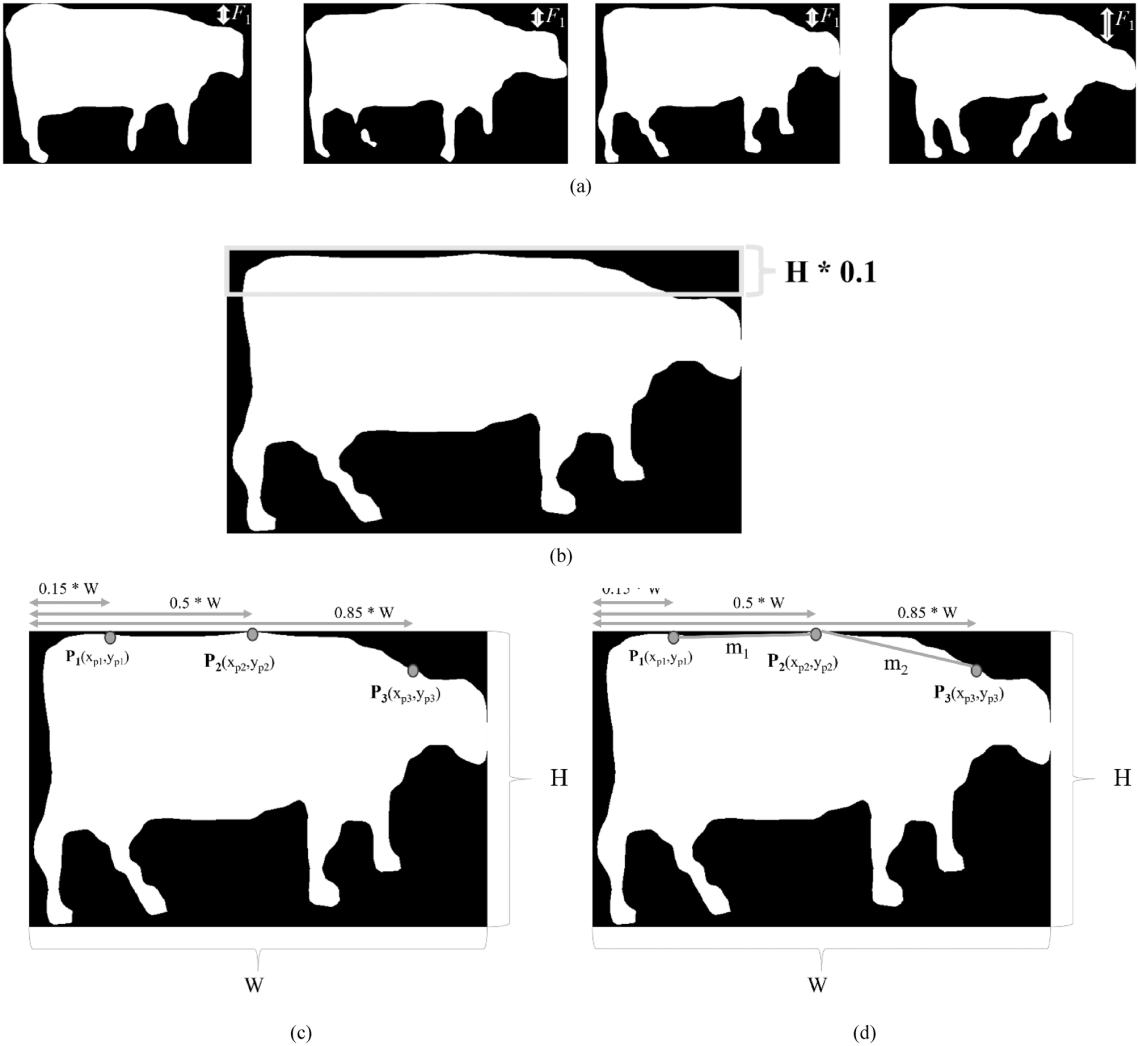
Feature extractions: (**a**) feature 1 (*f*_1_): distance between the upper bounding box and head region points. (**b**) Feature 2 (*f*_2_): the number of black pixels within the top 10% of the frame. (**c**) Feature 3 (*f*_3_): three points on the back curvature of the cattle. (**d**) Feature 4 (*f*_4_): slopes of the three points on the back curvature of the cattle.

These binary masks played a pivotal role in extracting pertinent features for the subsequent lameness classification process. Figure 11 offers a visual representation of sample binary mask images, each associated with different lameness scores. The distinctive characteristic lies in the shape of the cattle's back. While normal cattle exhibit a flat back structure, lame cattle display varying degrees of an arched back, ranging from mild to prominently arched. To extract meaningful features, the binary mask video sequence was initially subjected to a labelling process to identify the cattle regions. From these binary images, two key features were extracted^42^. The first feature involves measuring the vertical distance from the top border of the image to the head of cattle region. This feature holds significance in identifying lame cattle, as their head movement during walking tends to be either subtle or visibly pronounced. This process is depicted in Fig. 13a. By using the Eq. (3) the cattle ‘head distance value variance according to the height of cattle. To overcome this, the value of distance is quotient by each cattle’s height.

According to the paper^42^, the second feature (*F*_2_) revolves around calculating the area of the cattle back and the inclined head region. To compute this feature, the upper portion of the cattle region, spanning 10 percent of Height (H) of frame from the top edge, is cropped. The area ratio between the black region and the cattle object region is then determined using Eq. (4). The visual depiction of this process is presented in Fig. 13b. For feature (*F*_3_), the study employs an innovative feature extraction technique that utilizes three distinct points along the curvature of the cattle back. These key points are strategically located on the back, in the middle of the body, and on the neck. The objective of this technique is to construct a feature vector, as defined by Eq. (5), which is a cropped binary image of the cattle anatomical region shown in Fig. 13c.

3 $$F_{1} = y_{p3}$$

4 $$F_{2} = \frac{{ \# \left( {\text{black pixel}} \right)}}{{\text{W*H}}}$$

5 $$F_{3} = \frac{1}{H}\left( {y_{p1, } y_{p2, } y_{p3, } } \right)$$

6 $$F_{4} = \left( { \frac{{y_{p2} - y_{p1} }}{{x_{p2} - x_{p1} }}, \frac{{y_{p2} - y_{p3} }}{{x_{p2} - x_{p3} }}} \right)$$

In this study, we carefully identified three specific points on the curvature of cattle back within the cropped binary image. These points are designated as follows: Point 1 (P1): Located at 15% of the length of the cropped binary image width (W), point P1 corresponds to the back of the cattle. Point 2 (P2): Located at the midpoint (50%) of the length of the cropped binary image width (W), point P2 represents the midriff or midsection of the cattle. Point 3 (P3): This point signifies the head of the cattle and is situated at 85% of the cattle's length within the cropped binary image width (W). By employing this method, we aim to extract valuable information from the curvature of the cattle back, which ultimately contributes to the feature vector and facilitates the analysis of the cattle features. Another enhancement in this study, feature 4 illustrated in Fig. 13d, is the calculation of two different gradients associated with the three points along the curvature of the cattle back described in Eq. (6). This enhancement was introduced to further enhance the analysis of cattle curvature. Incorporating information on specific points and gradients has the potential to increase the accuracy and comprehensiveness of the study results. The strategic placement of these three identified points along the curvature of the cattle back serves an important purpose. Each of these points is meticulously placed to address a specific area of interest. This strategic placement facilitates targeted analysis and accurate feature extraction within the study. This innovative approach not only considers the spatial distribution of these points, but also the gradients that characterize the transitions between these points. This holistic approach is expected to provide more nuanced insights into cattle curvature, thereby contributing to a more refined understanding of cattle anatomy.

### Cattle lameness classification

In the Lameness classification phase, as the system needs correct ground truth dataset, we engaged a group of cattle experts to check all the cattle from farm manually and store the global identification and their lameness level sorted by the time frame that pass through the specific lane. The cattle experts check the cattle lameness level for the following dates as shown in Table 3.

**Table 3:** Number of cattle in each lameness level.

Lameness level	Lame 1	Lame 2	Lame 3	Lame 4
No of cattle	84	24	7	1

### Performance evaluation methods

7 $$Precision = \frac{{{\text{TP}}}}{{\text{TP + FP}}}$$

8 $$Recall = \frac{{{\text{TP}}}}{{\text{TP + FN}}}$$

where TP = true positive, FP = false positive, FN = false negative, precision is measuring the percentage of correct positive predictions among all predictions made; and recall is measuring the percentage of correct positive predictions among all positive cases.

### Ethics declarations

Ethical review and approval were waived for this study, due to no enforced nor uncomfortable restriction to the animals during the study period. The image data of calving process used for analysis in this study were collected by an installed camera without disturbing natural parturient behavior of animals and routine management of the farm.

## Experimental results

In the area of computer vision and machine learning, Python was primarily used as the programming language; the PyTorch framework was employed to build the deep learning components, including instance-segmentation for Detectron2; Scikit-learn was used for lameness classification of various classifiers was employed to implement the data. Additionally, data preprocessing tasks were performed using OpenCV, while data analysis and visualization were performed using NumPy, Pandas, and Matplotlib. This study covers cattle detection, tracking, and lameness classification as separate processes, and their performance metrics and results are calculated as follows.

### Cattle detection

During the cattle detection stage, our system leveraged an annotation dataset generated as cattle passed through the lane following the milk production process, occurring twice daily for each cattle. To facilitate the training of cattle detection, we specifically selected three different days from the dataset provided in Table 1. Out of the total dataset, we randomly extracted 3176 images (80%) for training purposes and reserved 794 images (20%) for validation as in Table 4. For the cattle detection model, we opted for the Mask RCNN architecture using the R-101-FPN-3 × configuration from COCO-Instance Segmentation. This choice was made to ensure robust segmentation and accurate identification of cattle instances within the images. The application of this algorithm resulted in the extraction of both cattle mask regions and corresponding bounding boxes, as demonstrated in Fig. 7. This approach allowed us to harness the power of deep learning and convolutional neural networks to detect cattle instances efficiently and effectively, aiding in the subsequent stages of our research aimed at cattle tracking and lameness classification.

**Table 4:** Training and validation images for detection.

#Images (100%)	#Train images (80%)	#Validation images (20%)
5458	4366	1092

In our evaluation process, we assess the performance of the detection model using three key types of Average Precision (AP) values, namely AP, AP50, and AP75, as outlined in Table 5. These metrics offer insights into the accuracy of both box and mask predictions. To ensure a comprehensive evaluation, we calculate the average precision over all Intersection over Union (IoU) thresholds (AP). Additionally, we focus on the AP values at IoU thresholds of 0.5 (AP50) and 0.75 (AP75), adhering to the COCO standard. These metrics provide a robust understanding of the model's ability to accurately predict cattle instances, catering to different levels of precision and IoU thresholds. Table 6, the accuracy of the testing detection results is presented for both morning and evening data spanning from January. The aggregated testing results reveal a commendable overall accuracy of 98.98%, as summarized in Table 6.

**Table 5:** Comparative evaluation of cattle detection performance on the validation dataset using average precision (AP) metrics for bounding boxes (BOX) and segmentation masks (Mask).

Box_AP_	Box_AP50_	Box_AP75_	Mask_AP_	Mask_AP50_	Mask_AP75_
94.31	99.53	99.534	87.75	99.53	99.53

**Table 6:** Evaluation metrics on January dataset for cattle detection by using Detectron2 (M = morning, E = evening, TP = true positive, TN = true negative, FP = false positive, FN = false negative).

Date	#Instances	#Correct instances (TP)	#Incorrect instances (FP)	#Miss instances (FN)	Recall (%)	Precision (%)	Accuracy (%)
3rd January 2023 (M)	9247	8855	392	0	100.00	95.76	95.76
3rd January 2023 (E)	8075	8000	75	0	100.00	99.07	99.07
5th January 2023 (M)	13,631	13,552	56	23	99.83	99.59	99.59
5th January 2023 (E)	8563	8511	35	17	99.80	99.59	99.59
6th January 2023 (M)	5059	4982	77	0	100.00	98.48	98.48
7th January 2023 (M)	5106	5106	0	0	100.00	100.00	100.00
10th January 2023 (M)	10,418	10,097	321	0	100.00	96.92	96.92
10th January 2023 (E)	10,302	10,228	74	0	100.00	99.28	99.28
11st January 2023 (M)	4476	4408	68	0	100.00	98.48	98.48
11st January 2023 (E)	6781	6770	11	0	100.00	99.84	99.84
12nd January 2023 (M)	5422	5405	17	0	100.00	99.69	99.69
12nd January 2023 (E)	10,852	10,814	38	0	100.00	99.65	99.65
13rd January 2023 (M)	9170	9001	169	0	100.00	98.16	98.16
13rd January 2023 (E)	15,368	14,797	571	0	100.00	96.28	96.28
14th January 2023 (M)	6074	6012	62	0	100.00	98.98	98.98
23rd January 2023 (M)	6668	6611	57	0	100.00	99.15	99.15
23rd January 2023 (E)	11,911	11,867	44	0	100.00	99.63	99.63
24th January 2023 (M)	12,546	12,450	96	0	100.00	99.23	99.23
24th January 2023 (E)	11,138	10,996	142	0	100.00	98.73	98.73
25th January 2023 (M)	7624	7587	37	0	100.00	99.51	99.51
25th January 2023 (E)	7972	7940	32	0	100.00	99.60	99.60
26th January 2023 (M)	6031	5963	68	0	100.00	98.87	98.87
26th January 2023 (E)	8967	8942	25	0	100.00	99.72	99.72
27th January 2023 (M)	11,064	11,032	32	0	100.00	99.71	99.71
27th January 2023 (E)	1870	1864	6	0	100.00	99.68	99.68
28th January 2023 (M)	15,619	15,422	197	0	100.00	98.74	98.74
29th January 2023 (M)	8168	8168	0	0	100.00	100.00	100.00
29th January 2023 (E)	9128	9065	63	0	100.00	99.31	99.31

**Figure 14:**
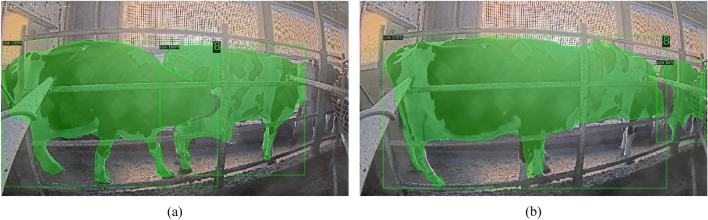
Cattle detection segmentation result (**a**) distinct regions (**b**) overlapping region.

Real-world cattle detection scenarios can present challenges, particularly in low-light environments. One such challenge arises when cattle are positioned close together, leading to overlapping instances. Standard segmentation algorithms may struggle to differentiate between individual cattle in these scenarios, resulting in a single merged region instead of two distinct ones as illustrated in Fig. 14a,b. This merged region can lead to two potential errors such as the system might misinterpret the merged region as abnormally large cattle, leading to an incorrect lameness detection and if the merged region's width falls below a minimum size threshold established for individual cattle, it could be excluded entirely. This would result in missed detections of individual cattle and potential lameness cases. To address the challenge of overlapping cattle and improve the accuracy of our system, we leverage the concept of minimum and maximum widths for cattle regions. We establish minimum and maximum width values based on prior analysis of cattle dimensions within our training dataset (described in Table 1). This analysis allows us to determine realistic cattle sizes within the specific breed and environment represented by the dataset. After the initial cattle region detection stage, the width of each detected region is analyzed. Cattle regions exceeding the maximum width threshold are discarded as potential errors caused by overlapping cattle. This eliminates unrealistically large, merged cattle regions that likely represent multiple cattle.

**Figure 15:**
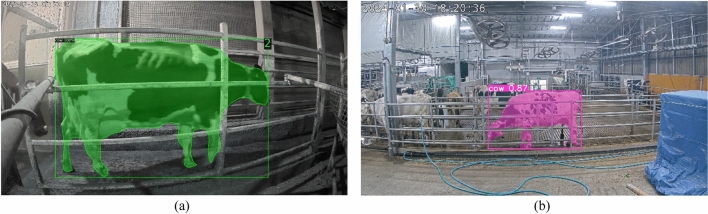
Cattle Detection Result from multiple farms (**a**) dataset from Hokuren Kunneppu (**b**) dataset from Sumiyoshi.

We evaluated the cattle detection component of our system using data from another farm, "Sumiyoshi." This testing across different farms demonstrates the system's ability to handle variations in cattle appearance and farm environment. Figure 15a showcases cattle detection results on our and Fig. 15b highlights results on dataset from "Sumiyoshi" farm, demonstrating the system's generalizability to unseen variations. This high accuracy level underscores the effectiveness and reliability of the system's detection capabilities across various instances and time frames. When comparing the performance evaluation of our main cattle detection algorithm with the current most popular real-time object detection and image segmentation model YOLOv8 in Table 7 using 2023 March 14 evening dataset, we found that YOLOv8 achieved a higher accuracy of 99.92% compared to our custom detection algorithm. However, there were instances of missed detection (False Negative) in some frames. On the other hand, the detectron2 detection had no missed detection, but there were some instances of wrongly detecting humans as cattle.

**Table 7:** Comparison of performance evaluation between YOLOv8x and Detectron2 (TP = true positive, TN = true negative, FP = false positive, FN = false negative).

Model	#Instances	#Correct (TP)	#Wrong (FP)	(TN)	(FN)	Recall (%)	Precision (%)	Accuracy (%)
YOLOv8x	15,305	15,224	12	0	69	99.92	99.55	99.92
Detectron2	15,305	14,466	839	0	0	94.52	100.00	94.52

**Figure 16:**
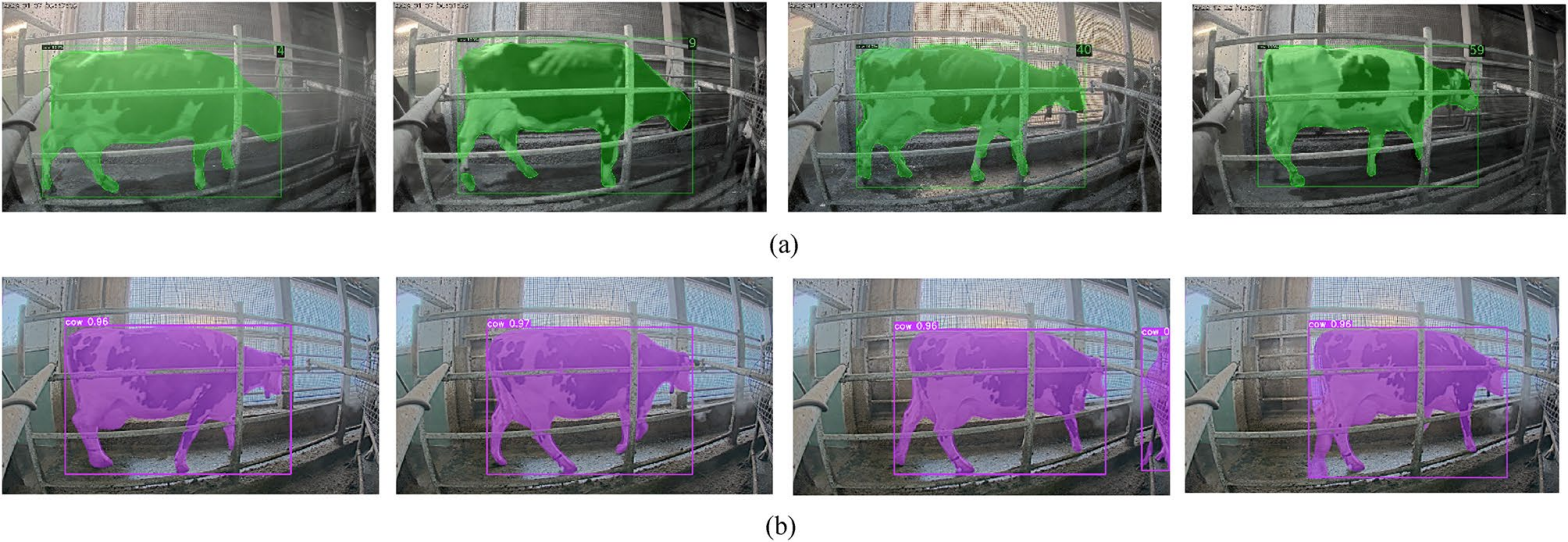
Cattle detection result (**a**) by using Detectron2 (**b**) by using YOLOv8.

Therefore, we recommend using our main detection algorithm, detectron2, as it removes the wrongly detected human regions by calculating the area of the detected object and comparing it to a constant threshold. Some of the detection results are shown in Fig. 16. Detection by using detectron2 is described in Fig. 16a and detection by using YOLOv8x is as depicted in Fig. 16b.

### Cattle tracking

The process of tracking was a sophisticated technique that capitalizes on bounding boxes, which are created during the Detection phase. This phase was of paramount importance as it pertains to the segregation of the cattle based on their distinct LocalID, a process which is clearly illustrated in Fig. 10. Once this is accomplished, each frame is meticulously preserved and stored in a separate folder, a step that is crucial for future computations of lameness, as depicted in Fig. 11. Table 8 provides an in-depth analysis of the outcomes of cattle tracking for the comprehensive dataset that was compiled in the month of January. Our Customized Tracking Algorithm (CTA) emerges as an extremely efficient solution, providing a compelling alternative to other prevalent tracking algorithms in terms of both runtime and complexity. The detailed workings of this algorithm are outlined in Algorithm 1. We optimized the CTA to effectively address and overcome the various challenges associated with tracking. The results of these efforts are evident in its performance, as it achieved a remarkable accuracy rate of 100.00% in most cases. On average, the accuracy of the CTA was an impressive 99.5% when applied to the January dataset. On an average day, we observed that approximately 58 cattle would traverse the lane that connects the milking parlor to the cattle barn. This daily movement of cattle is an important aspect of our tracking and data collection process.

**Table 8:** Evaluation metrics on January dataset for cattle tracking by using custom tracking algorithm (M = morning, E = evening).

Date	#Cattle	#Correct (TP)	#Wrong (FP)	Accuracy (%)
3rd January 2023 (M)	62	62	0	100.00
3rd January 2023 (E)	56	56	0	100.00
5th January 2023 (M)	64	63	1	98.44
5th January 2023 (E)	65	64	1	98.46
6th January 2023 (M)	64	64	0	100.00
7th January 2023 (M)	42	41	1	97.62
10th January 2023 (M)	56	55	1	98.21
10th January 2023 (E)	56	56	0	100.00
11st January 2023 (M)	56	56	0	100.00
11st January 2023 (E)	63	63	0	100.00
12nd January 2023 (M)	56	56	0	100.00
12nd January 2023 (E)	58	57	1	98.28
13rd January 2023 (M)	66	65	1	98.48
13rd January 2023 (E)	54	54	0	100.00
14th January 2023 (M)	64	64	0	100.00
23rd January 2023 (M)	63	63	0	100.00
23rd January 2023 (E)	64	64	0	100.00
24th January 2023 (M)	55	54	1	98.18
24th January 2023 (E)	56	56	0	100.00
25th January 2023 (M)	58	58	0	100.00
25th January 2023 (E)	58	58	0	100.00
26th January 2023 (M)	52	52	0	100.00
26th January 2023 (E)	62	62	0	100.00
27th January 2023 (M)	27	27	0	100.00
27th January 2023 (E)	62	62	0	100.00
28th January 2023 (M)	41	41	0	100.00
29th January 2023 (M)	62	61	1	98.38
29th January 2023 (E)	62	62	0	100.00

### Feature extraction

Ground truth data, collected from farm evaluations by a lameness expert, links each cow's lameness level to its unique ear tag ID. This data serves as a benchmark for evaluating our proposed model's performance. Table 9 details the distribution of lameness levels within the cattle population. As shown, 84 cattle are classified as level 1 (no lameness), while levels 2 and 3 represent stages of early lameness. Notably, our system identified only one cow exhibiting level 4 lameness. We have extracted four features [*F*_1_*, F*_2_*, F*_3_*, F*_4_] and their extraction methods utilizing Eqs. 3–6. We created a cattle lameness classification dataset using over 1000 images shown in Table 10. Out of these, 690 images are used for training and 341 images for testing. This is done to assess the efficiency and effectiveness of the extracted features in lameness classification calculations. In this dataset, 694 frames are labeled as “No lameness” and 337 frames as “lameness”.

**Table 9:** Lameness level ground truth.

Lameness level	Lame 1	Lame 2	Lame 3	Lame 4
No of cattle	84	24	7	1

**Table 10:** Training and testing images for feature extraction.

#Total images	#Training images	#Testing images
1031	690	341

**Figure 17:**
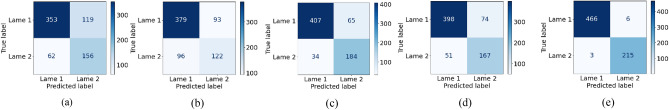
Training results using SVM with different feature sets (**a**) feature 1 (**b**) feature 2 (**c**) feature 3 (**d**) feature 4 (**e**) by combining all the four features (lame 1 = lameness level 1, lame 2 = lameness level 2).

**Figure 18:**
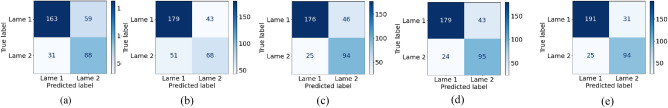
Testing Results using SVM with different feature sets (**a**) feature 1 (**b**) feature 2 (**c**) feature 3 (**d**) feature 4 (**e**) by combining all the four features (lame 1 = lameness level 1, lame 2 = lameness level 2).

Selecting and evaluating informative features presents a significant challenge for our proposed system. Each feature extracted from the dataset (Table 10) is evaluated using a Support Vector Machine (SVM) classifier. The training set classification results are presented in Fig. 17a–d. As observed, each individual feature demonstrates some level of effectiveness for lameness classification. Notably, Fig. 17e demonstrates that combining all features into a feature vector yields superior performance compared to using individual features. This combined feature approach is further validated on the testing dataset using the SVM classifier, as shown in Fig. 18a–d for individual features and Fig. 18e for the combined feature vector. The results confirm that utilizing all features collectively leads to a more efficient classification process compared to relying on individual features. Building upon the feature analysis, the subsequent section on “Cattle Lameness Classification” will employ all four features (*F*_1_*, F*_2_*, F*_3_*, and F*_4_) combined into a feature vector.

### Cattle lameness classification

Traditional lameness assessment often relies on subjective visual inspection by experts, a process susceptible to inconsistencies and time delays. Machine learning (ML) algorithms offer a compelling alternative by leveraging extracted features from video recordings to objectively analyze cattle movement patterns. This study explores the efficacy of three established ML algorithms: Support Vector Machines (SVM), Random Forests (RF), and Decision Trees (DT), along with AdaBoost and Stochastic Gradient Descent (SGD), for automated cattle lameness classification based on image-derived features.

We prioritized optimizing the classification process for real-time application and reliability. The proposed system was evaluated using the January dataset (Table 11). Notably, AdaBoost achieved the highest overall average accuracy (77.9%), followed by DT (75.32%), SVM (75.20%), and Random Forest (74.9%). While AdaBoost, SVM, DT, and RF exhibited accuracy exceeding 80% on specific dates within the January dataset, AdaBoost demonstrably optimized average accuracy across the entire dataset. Conversely, SGD did not achieve the highest accuracy levels.

**Table 11:** Comparative performance analysis of machine learning classifiers for cattle lameness detection on the January dataset (M = morning, E = evening).

Date	SVM	RF	DT	AdaBoost	SGD
3rd January 2023 (M)	75.81	67.74	70.97	80.65	45.16
3rd January 2023 (E)	78.57	76.79	78.57	82.14	51.79
5th January 2023 (M)	71.88	71.88	60.94	73.44	42.19
5th January 2023 (E)	78.46	81.54	83.08	81.54	41.54
6th January 2023 (M)	78.12	68.75	76.56	73.44	40.62
7th January 2023 (M)	71.43	69.05	59.52	69.05	42.86
10th January 2023 (M)	83.93	78.57	85.71	80.36	50.00
10th January 2023 (E)	80.36	71.43	75.00	78.57	51.79
11st January 2023 (M)	69.64	67.86	76.79	75.00	44.64
11st January 2023 (E)	71.43	73.21	71.43	76.79	51.79
12nd January 2023 (M)	71.43	79.37	79.37	79.37	46.03
12nd January 2023 (E)	80.36	80.36	78.57	80.36	51.79
23rd January 2023 (M)	71.43	76.19	80.95	80.95	50.79
23rd January 2023 (E)	75.00	78.12	76.56	75.00	50.00
24th January 2023 (M)	71.7	73.58	77.36	77.36	56.60
24th January 2023 (E)	67.86	73.21	67.86	73.21	58.93
25th January 2023 (M)	73.21	71.43	76.79	78.57	35.71
25th January 2023 (E)	81.36	81.36	76.27	79.66	47.46
26th January 2023 (E)	76.92	82.69	78.85	84.62	51.92

## Conclusion

This study presents a novel cattle detection and tracking approach leveraging the capabilities of the Detectron2 model. We evaluate its performance against the state-of-the-art (SOTA) YOLOv8 model. Our system demonstrates exceptional performance in cattle region detection, further enhanced by incorporating Intersection over Union (IoU) calculations with frame holding for robust and accurate tracking. Notably, on the validation dataset, the proposed system achieves an accuracy exceeding 90% for both detection and tracking tasks. Future work aims to leverage diverse deep learning methodologies to refine the system's accuracy and robustness. Specifically, we will address challenges such as accurately identifying and integrating overlapping cattle instances across detection lanes. The core objective of this study is to explore lameness-inducing factors and methods for early detection. Prior to lameness classification, the system utilizes instance segmentation algorithms to detect cattle regions and extract mask values, which serve as crucial data for feature extraction. This approach integrates image processing techniques with deep learning methods for robust detection and tracking. Extracted features (*F*_1_, *F*_2_, *F*_3_, *F*_4_) are subsequently combined into feature vectors and classified using established machine learning algorithms, including SVM, RF, DT, AdaBoost, and SGD. Notably, AdaBoost achieved an optimized average accuracy of 77.9% on the January testing dataset. Our commitment to continuous innovation and excellence drives us. We envision future iterations integrating cutting-edge deep learning algorithms for lameness classification, followed by a comprehensive comparative analysis.

## Data Availability

The datasets generated during and/or analysed during the current study are available from the corresponding author on reasonable request.
